# Novel Insights into Energy Storage Mechanism of Aqueous Rechargeable Zn/MnO_2_ Batteries with Participation of Mn^2+^

**DOI:** 10.1007/s40820-019-0278-9

**Published:** 2019-06-06

**Authors:** Yongfeng Huang, Jian Mou, Wenbao Liu, Xianli Wang, Liubing Dong, Feiyu Kang, Chengjun Xu

**Affiliations:** 1 Shenzhen Geim Graphene Center, Graduate School at Shenzhen, Tsinghua University, Shenzhen, 518055 People’s Republic of China; 2 State Key Laboratory of New Ceramics and Fine Processing, School of Materials Science and Engineering, Tsinghua University, Beijing, 100084 People’s Republic of China

**Keywords:** Zinc-ion battery, MnO_2_ cathode, Energy storage mechanism, Phase evolution

## Abstract

Aqueous rechargeable Zn/MnO_2_ zinc-ion batteries (ZIBs) are reviving recently due to their low cost, non-toxicity, and natural abundance. However, their energy storage mechanism remains controversial due to their complicated electrochemical reactions. Meanwhile, to achieve satisfactory cyclic stability and rate performance of the Zn/MnO_2_ ZIBs, Mn^2+^ is introduced in the electrolyte (e.g., ZnSO_4_ solution), which leads to more complicated reactions inside the ZIBs systems. Herein, based on comprehensive analysis methods including electrochemical analysis and Pourbaix diagram, we provide novel insights into the energy storage mechanism of Zn/MnO_2_ batteries in the presence of Mn^2+^. A complex series of electrochemical reactions with the co-participation of Zn^2+^, H^+^, Mn^2+^, SO_4_^2−^, and OH^−^ were revealed. During the first discharge process, co-insertion of Zn^2+^ and H^+^ promotes the transformation of MnO_2_ into Zn_*x*_MnO_4_, MnOOH, and Mn_2_O_3_, accompanying with increased electrolyte pH and the formation of ZnSO_4_·3Zn(OH)_2_·5H_2_O. During the subsequent charge process, Zn_*x*_MnO_4_, MnOOH, and Mn_2_O_3_ revert to α-MnO_2_ with the extraction of Zn^2+^ and H^+^, while ZnSO_4_·3Zn(OH)_2_·5H_2_O reacts with Mn^2+^ to form ZnMn_3_O_7_·3H_2_O. In the following charge/discharge processes, besides aforementioned electrochemical reactions, Zn^2+^ reversibly insert into/extract from α-MnO_2_, Zn_*x*_MnO_4_, and ZnMn_3_O_7_·3H_2_O hosts; ZnSO_4_·3Zn(OH)_2_·5H_2_O, Zn_2_Mn_3_O_8_, and ZnMn_2_O_4_ convert mutually with the participation of Mn^2+^. This work is believed to provide theoretical guidance for further research on high-performance ZIBs.

**Electronic supplementary material:**

The online version of this article (10.1007/s40820-019-0278-9) contains supplementary material, which is available to authorized users.

## Highlights

- Pourbaix diagram of Mn–Zn–H_2_O system was used to analyze the charge–discharge processes of Zn/MnO_2_ batteries.
- Electrochemical reactions with the participation of various ions inside Zn/MnO_2_ batteries were revealed.
- A detailed explanation of phase evolution inside Zn/MnO_2_ batteries was provided.

**Electronic supplementary material:**

The online version of this article (10.1007/s40820-019-0278-9) contains supplementary material, which is available to authorized users.

## Graphical abstract

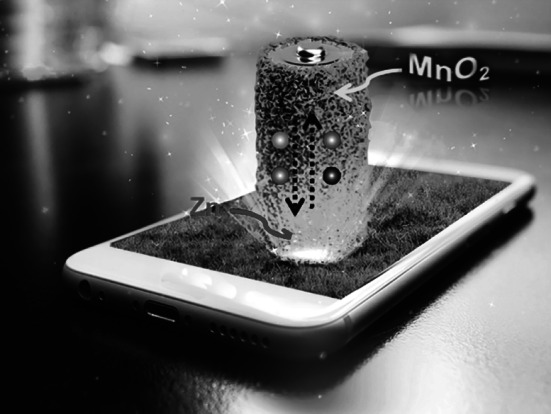

## Introduction

Lithium-ion batteries (LIBs) have been widely used in consumer electronics due to high energy density, portability, and some other merits [1–4], whereas their security concerns and high cost restrict their large-scale applications in stationary grid storage and electric vehicles [5, 6]. Therefore, much attention has been paid to seek safe, eco-friendly, low-cost, and high-performance battery systems [6, 7]. Aqueous rechargeable zinc-ion batteries (ZIBs) are developed as a battery system, in which low-cost, non-toxic, and naturally abundant zinc metal is used as an anode and environment-friendly neutral aqueous Zn^2+^-containing solution serves as electrolyte [8]. In recent years, a series of high-performance cathode materials for ZIBs have also been studied such as Prussian blue analog [9–11], vanadium oxides [12–19], manganese oxides [20–28], and some metal sulfides [29–31]. Among these materials, MnO_2_ is particularly concerned for its high theoretical specific capacity, low cost, eco-friendliness, and diverse crystallographic polymorphs (e.g., *α*-MnO_2_, δ-MnO_2_, and γ-MnO_2_) [27, 28, 32].

Many efforts have been made to reveal the energy storage mechanisms of Zn/MnO_2_ ZIBs. Up to now, three types of energy storage mechanisms were proposed, including (i) Zn^2+^ insertion/extraction into/from MnO_2_ [8, 33–36], (ii) conversion between MnO_2_ and MnOOH with the participation of H^+^ [37], and (iii) co-insertion of H^+^ and Zn^2+^ [38]. Mechanisms (i) and (ii) explain the formation of ZnMn_2_O_4_ and MnOOH as discharging products on MnO_2_ cathode in ZIBs, respectively, while cannot explain that there are two redox processes during one charge/discharge cycle of ZIBs. Mechanism (iii) seems to be capable of explaining the coexistence of ZnMn_2_O_4_ and MnOOH as discharging products on MnO_2_ cathode, but deeper analysis will find that it is not accurate: The mechanism deems that potential of Zn^2+^ insertion is lower than that of H^+^ insertion (this means that MnOOH forms before ZnMn_2_O_4_ once the battery discharge process begins), being conflicted to the experimental result that MnOOH appears latter than ZnMn_2_O_4_. In short, the current mechanisms are unsatisfactory to explain genuine charge/discharge process in ZIBs, mainly because they were proposed based on a simplistic view that the insertion of Zn^2+^ and H^+^ and the phase change from MnO_2_ to ZnMn_2_O_4_ or MnOOH are highly reversible. Furthermore, to achieve satisfactory cyclic stability and rate performance of the Zn/MnO_2_ ZIBs, Mn^2+^ ions are always introduced in the electrolyte [37]. However, electrochemical reactions inside the ZIBs become more complicated in such cases, thus corresponding energy storage mechanism has not been clearly revealed. Therefore, it is necessary to re-examine the thermodynamic and kinetic characteristics of Zn/MnO_2_ ZIBs to propose a reasonable Zn^2+^ storage mechanism.

In fact, for the active materials in aqueous ZIBs and some other rechargeable aqueous batteries, their structure and phase generally undergo complex changes during charge/discharge processes (e.g., the active materials can interact with not only metal ions, but also H^+^, OH^−^, and water molecules) [39]. This is an important reason why the energy storage mechanism of MnO_2_ cathode in ZIBs is still inconclusive [40–43]. Besides general experimental techniques such as cyclic voltammetry (CV) and galvanostatic charge–discharge (GCD) tests, Pourbaix diagram (E-pH diagram) has been widely used to study electrochemical reactions in aqueous solution [44–47]. The electrochemical reductive products of active materials can be predicted according to the thermodynamics, which is beneficial for us to understand the charge/discharge process. Therefore, we combined experimental methods with the E-pH diagram of the Mn–Zn–H_2_O system together to comprehensively analyze the charge/discharge processes of MnO_2_ cathode in ZIBs and tried to reveal the authentic energy storage mechanism.

Herein, based on comprehensive analysis methods including electrochemical analysis and E-pH diagram, etc., we provide novel insights into the energy storage mechanism of Zn/MnO_2_ batteries with the co-participation of Zn^2+^, H^+^, Mn^2+^, SO_4_^2−^, and OH^−^. During the first discharge process, co-insertion of Zn^2+^ and H^+^ promotes the transformation of MnO_2_ into Zn_*x*_MnO_4_, MnOOH, and Mn_2_O_3_, accompanying with increased electrolyte pH and the formation of ZnSO_4_·3Zn(OH)_2_·5H_2_O (noted as “BZSP”). During the subsequent charge process, Zn_*x*_MnO_4_, MnOOH, and Mn_2_O_3_ revert to α-MnO_2_ with the extraction of Zn^2+^ and H^+^, while BZSP reacts with Mn^2+^ to form ZnMn_3_O_7_·3H_2_O. In the following charge/discharge processes, besides aforementioned electrochemical reactions, Zn^2+^ reversibly inserts into/extract from α-MnO_2_, Zn_*x*_MnO_4_, and ZnMn_3_O_7_·3H_2_O hosts, and BZSP, Zn_2_Mn_3_O_8_, and ZnMn_2_O_4_ convert mutually with the participation of Mn^2+^. This work is believed to provide theoretical guidance for further research on high-performance ZIBs.

## Experimental

### Material Synthesis

MnO_2_ cathode material was synthesized through a chemical co-precipitation method. One hundred and fifty milliliters of 0.1 M MnSO_4_ aqueous solution was dropped into 100 mL of KMnO_4_ (0.1 M) solution under magnetic stirring, followed by continuous stirring for 6 h at room temperature. The resulting precipitate was filtered, washed repeatedly with deionized water, and dried at 80 °C for 12 h. The obtained sample was thoroughly ground in an agate mortar and then annealed at 400 °C for 5 h in air atmosphere. Note that we applied the heat treatment to improve the crystalline of MnO_2_ because this makes it easier for us to use X-ray diffraction (XRD) and transmission electron microscopy (TEM) to detect the phase evolution of MnO_2_ cathode during charge/discharge processes.

### Electrochemical Characterizations

The cathode was prepared by coating a mixture paste of 70 wt% of MnO_2_ powder, 20 wt% of acetylene black, and 10 wt% of LA133 binder on a stainless steel foil and dried overnight under vacuum conditions at 80 °C. In the prepared cathode, the mass loading of MnO_2_ is around 1 mg cm^−2^. Zn/MnO_2_ ZIBs were assembled based on MnO_2_ cathode, metallic Zn foil anode, air-laid paper separator, and zinc salt solution electrolyte (2 M ZnSO_4_ or 2 M ZnSO_4_ +0.5 M MnSO_4_ solution).

The assembled ZIBs were kept more than 4 h before electrochemical measurements. The CV and GCD tests were performed on a Bio-logic VMP3 multichannel electrochemical station and a Land CT2001 battery tester, respectively. CV tests of the prepared MnO_2_ cathode were also carried out in a three-electrode system, in which a platinum plate as the counter electrode, a saturated calomel electrode (SCE) as the reference electrode, and 50 mL electrolyte was applied.

### Material Characterizations

Microstructure and composition were characterized by XRD (Rigaku 2500) with Cu-K*α* radiation operating at 40 kV and 100 mA within an angle range of 10° to 70° at a scan speed of 5° min^−1^. Micro-morphology was observed by field emission scanning electron microscopy (SEM, Zeiss Supra 55) and TEM (Tecnai G2 F30). Element content in electrodes and electrolytes was analyzed by inductively coupled plasma atomic emission spectrometry (ICP-AES).

## Results and Discussion

### Characterization of MnO_2_

**Fig. 1:**
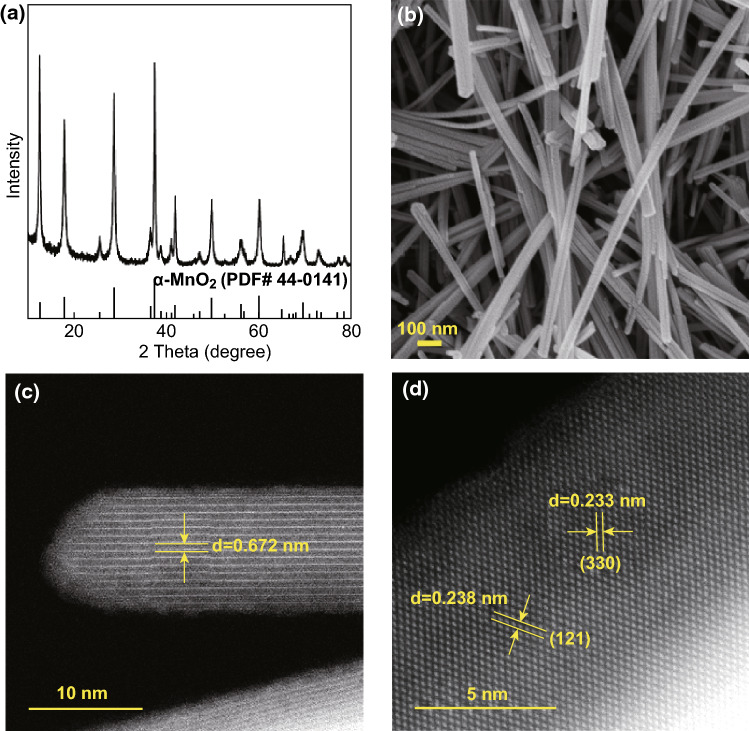
**a** XRD pattern, **b** SEM image, **c** STEM, and **d** HRSTEM images of the synthesized MnO_2_ nanorods

The MnO_2_ material used in this work was synthesized through a chemical co-precipitation method. XRD pattern and micro-morphology observations in Fig. 1a–c show that the synthesized MnO_2_ powder is crystalline α-MnO_2_ nanorod with a diameter of 10–60 nm and length of several hundred nanometers. From the XRD pattern in Fig. 1a, it seems that the strongest peak is the one at ~ 12.8°, but if the background is taken into account, the strongest peak is still the one at ~ 37.5°, which matches well with the α-MnO_2_ (PDF# 44-0141). In addition, since the as-prepared sample is nanobelts, (110) plane (corresponding to the diffraction peak at ~ 12.8°) is considered as preferred orientation, thus leading to high diffraction intensity. A similar phenomenon was also observed for some other MnO_2_ nanomaterials [38]. From the high-resolution TEM (HRTEM) image in Fig. 1d, the crystal planes (121) and (330) of the α-MnO_2_ with a corresponding interplanar spacing of 0.238 nm and 0.233 nm respectively are observed, and high crystallinity of the as-synthesized α-MnO_2_ sample is also confirmed.

### Electrochemical Analysis

**Fig. 2:**
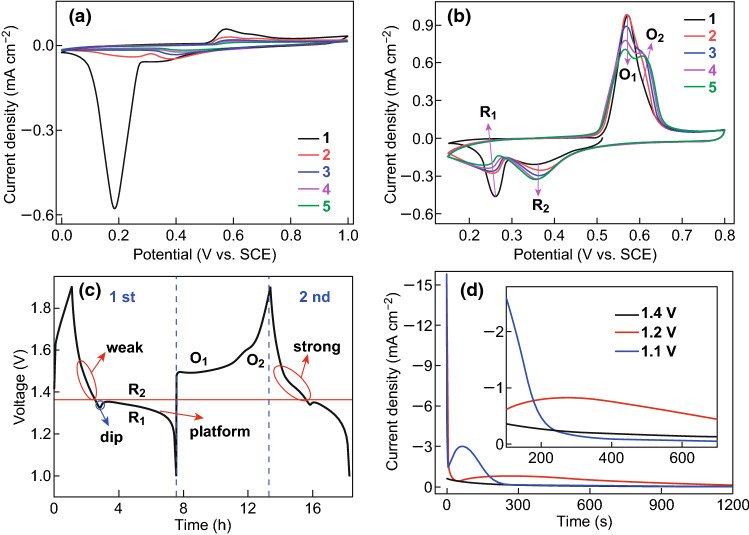
Electrochemical behaviors of the prepared cathodes: **a** CV curves in 50 mL 2 M ZnSO_4_ solution (first to fifth cycles); **b** CV (first to fifth cycles) and **c** GCD curves in 50 mL 2 M ZnSO_4_ + 0.5 M MnSO_4_ solution; **d** constant voltage discharge at various voltages (1.4, 1.2, and 1.1 V) and local magnification diagram (the inset)

We first studied the electrochemical behaviors of MnO_2_ cathode in two different electrolytes, including 50 mL 2 M ZnSO_4_ (Fig. 2a) and 50 mL 2 M ZnSO_4_ + 0.5 M MnSO_4_ mixture solution (Fig. 2b–d). The capacity and rate performance of the MnO_2_ cathode in ZnSO_4_ + MnSO_4_ electrolyte are exhibited in Fig. S1. Note that MnO_2_ cathode would dissolve in ZnSO_4_ electrolyte during charge/discharge processes, as detected by ICP-AES tests in Table S1. (This has also been pointed out in previous researches.) [37] With the addition of Mn^2+^ in the electrolyte, the redox peaks in CV curves (except for the 1st CV cycle) in Fig. 2a, b become more obvious, and meanwhile, the gap between oxidation peak and reduction peak becomes smaller, which indicates that the reversibility of electrochemical process gets better. In Fig. 2a, the reduction peak is much stronger than the oxidation peak in the first cycle, which means that discharge products cannot be electrochemically oxidized completely. By contrast, the intensity of the reduction peak is close to that of the oxidation peak in Fig. 2b. Above phenomenon suggests that Mn^2+^ concentration in electrolyte plays a crucial role in the first discharge/charge process [48]. There are two pairs of redox peaks when the electrode discharges/charges in the 2 M ZnSO_4_ + 0.5 M MnSO_4_ electrolyte (Fig. 2b). The reduction peaks at low and high potentials are denoted as R_1_ and R_2_, respectively, and the oxidation peaks at low and high potentials are denoted as O_1_ and O_2_, respectively. Considering that electro-deposition of Mn^2+^ will occur only when the cathode potential reaches about 0.8 V versus SCE at the constant current of 0.1 A cm^−2^ (Fig. S2), the oxidation reactions of O_1_ and O_2_ ranging from 0.5 to 0.65 V versus SCE (Fig. 2b) are not caused by the electro-deposition of Mn^2+^. This confirms that the preclusion of MnO_2_ dissolution by Mn^2+^ in the electrolyte should be the dominant reason for good reversibility of discharge/charge processes in 2 M ZnSO_4_ + 0.5 M MnSO_4_ electrolyte.

There is a dip and a platform in the initial GCD curve (Fig. 2c) and the reaction type of R_1_ (at about 1.2 V) and R_2_ (at about 1.4 V) are further studied by the constant voltage discharge test (Fig. 2d). The current changes greatly when the battery is discharged at 1.2 V at which R_1_ will happen, and it keeps almost flat when discharged at 1.4 V at which R_2_ will occur. This indicates that a heterogeneous reaction occurs during R_1_ and a homogeneous reaction occurs during R_2_. Such a heterogeneous reaction between solid phases accompanying with nucleation process and electro-crystallization process will cause the formation of the dip and steep curve in the discharge curve in Fig. 2c [49]. With the increasing CV cycles (Fig. 2b), the peak current intensity of R_1_ and O_1_ is getting weaker, while the peak current intensity of R_2_ and O_2_ becomes stronger. Therefore, the redox reactions R_1_/O_1_ and R_2_/O_2_ are more likely to be independent of each other. That the initial process differs from the subsequent one can also be seen from Fig. 2c (the red circle). R_2_ is weaker in the initial discharge process than that in the second one, which indicates that a new phase may generate as active materials.

### Phase Evolution of Cathode in the Initial Discharge Process

ex situ XRD tests of the cathodes at different charge/discharge states support were performed. As shown in Fig. S3, when the cathode is initially discharged from 1.9 to 1.4 V, no new phase produces, while the cathode is further discharged to 1.0 V, several new diffraction peaks occur, indicating the appearance of new phases. In the charging process, some diffraction peaks cannot be detected, which means the disappearance of some phases. After 100 charges/discharge cycles, the XRD pattern is not in conformity with the XRD patterns of the cathode at the original state and fully charged state in the 1st charge process. These demonstrate that the cathode undergoes a complicated phase evolution. In the following, phase evolution of MnO_2_ cathodes during the 1st discharge process, the 1st charge process and subsequent discharge/charge processes were investigated in detail.

**Fig. 3:**
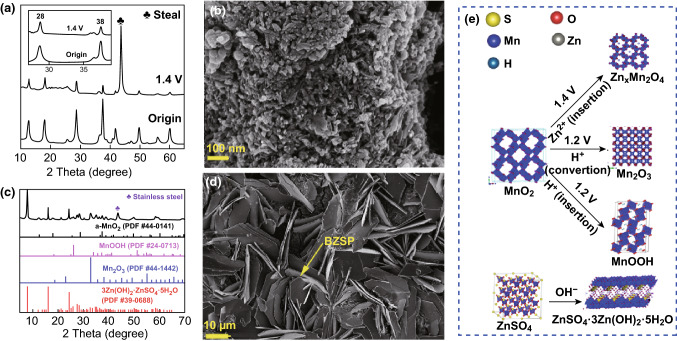
The results of the cathode which is constant current discharged to 1.4 V and then constant voltage discharged at 1.4 V in 2 M ZnSO_4_ + 0.5 M MnSO_4_ electrolyte for 2 h: **a** XRD pattern and locally enlarged image (the inset); **b** SEM image of the surface of the cathode. Another cathode which is constant current discharged to 1.0 V: **c** XRD pattern; **d** SEM image of the surface of the cathode; **e** phase evolution of cathode during the first discharge process

To investigate phases evolution of cathode in the initial discharge process in 2 M ZnSO_4_ + 0.5 M MnSO_4_ electrolyte, R_1_ and R_2_ reactions (as defined in Fig. 2, the same hereinafter) are separately studied. Only R_2_ occurs when the voltage of the battery is above 1.4 V. No new phase produces are seen from the XRD pattern (Fig. 3a). Nevertheless, characteristic peaks of the MnO_2_ active material such as the peaks at 28° and 38° shift (inset of Fig. 3a), which is attributed to the change of the layer spacing of MnO_2_. This means that the active material undergoes structure change. The nanorods become shorter (Fig. 3b) which is greatly different from that of the as-prepared material (Fig. 1b). Energy dispersive spectrometer (EDS) analysis in Fig. S4 suggests that the molar ratio of Zn, Mn, and S is approximately 1:16:0. To exclude the influence of electrolyte’s absorption, we immersed the electrodes in 2 M ZnSO_4_ for 48 h and washed several times with deionized water. Neither Zn nor S element is found in the SEM–EDS result (Fig. S5). Thus, the existence of Zn in the cathode when discharging to 1.4 V is caused by the insertion of zinc ions in MnO_2_, instead of zinc ion adsorption on the cathode surface. In other words, zinc ion insertion happens when the voltage of the Zn/MnO_2_ battery is above 1.4 V. The process of zinc-ion insertion in MnO_2_ can be written as:

1 $$ {\text{MnO}}_{2} + x{\text{Zn}}^{2 + } + 2x{\text{e}}^{ - } \to {\text{Zn}}_{x} {\text{MnO}}_{2} $$

**Fig. 4:**
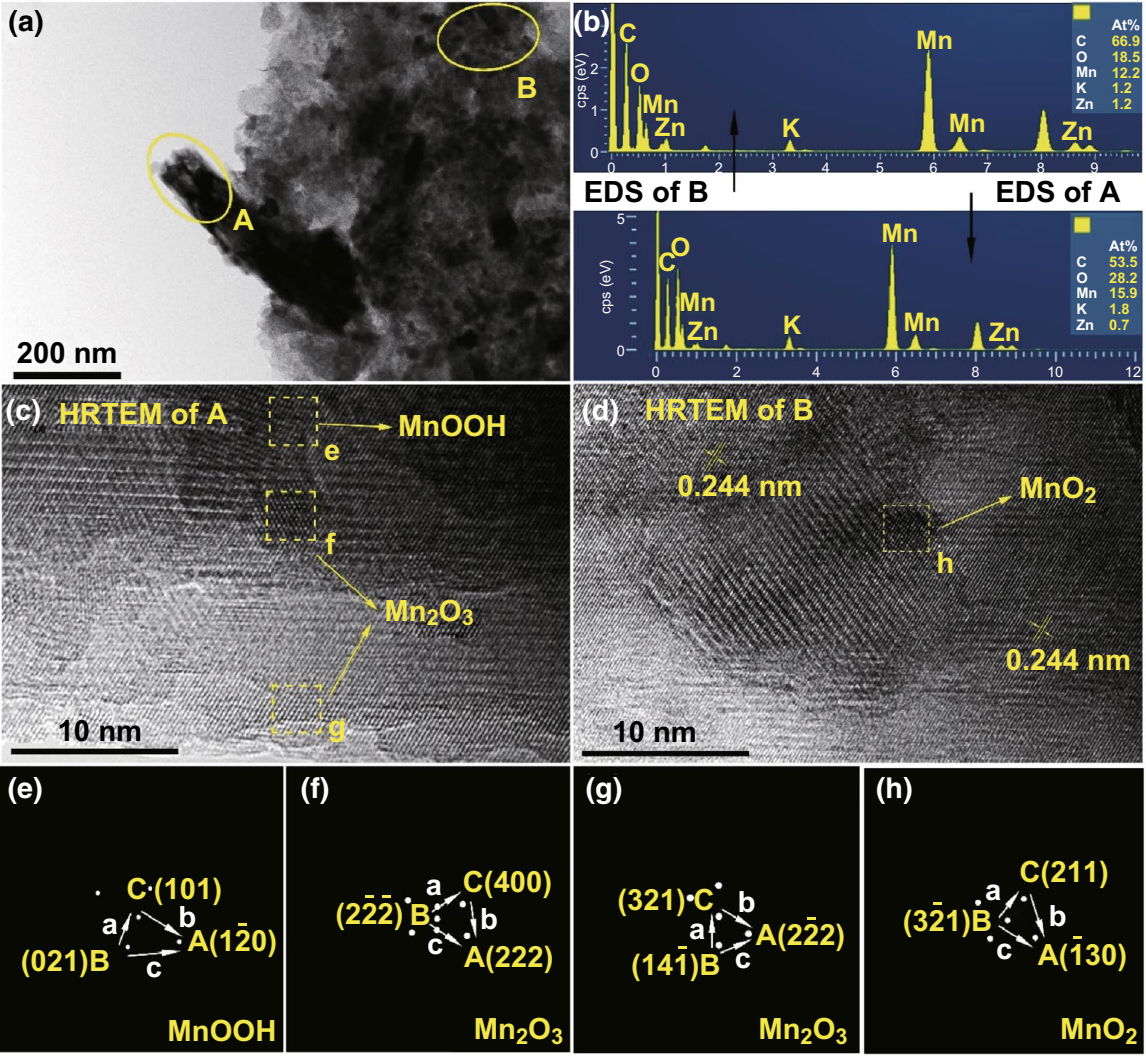
TEM, HRTEM image, and TEM-EDS spectrum of the sample constant current discharge to 1.0 V: **a** TEM image **b** TEM-EDS spectrum result of region A and B marked in **a**. **c, d** HRTEM image of region A and B marked in Fig. 4a, respectively. **e, f** FFT of the region marked by a yellow dash in **c** and **d**, respectively

R_1_ reaction is then studied. When the battery is discharged to 1.2 V and then to the 1 V at constant current several new phases (XRD patterns in Fig. 3c) appear, which are confirmed as BZSP (PDF#39-0688), α-MnOOH (PDF#24-0713), and α-Mn_2_O_3_ (PDF#44-1442). There are many large hexagonal nanosheets in the SEM image (Fig. 3d). The Zn, O, and S element distribute evenly over the whole hexagonal nanosheets (Fig. S6b, c, e, f). And the atomic proportion of Zn to S is about 4:1 from the SEM–EDS results (Fig. S6d). Combined with the XRD result, we conclude that the hexagonal nanosheets are BZSP. The structure evolution of cathode in the first discharge is shown in Fig. 3e. The existence of α-MnOOH and α-Mn_2_O_3_ is further demonstrated by HRTEM (Fig. 4). The α-Mn_2_O_3_ is semi-coherent with the α-MnOOH phase (Fig. 4c). These substances and their reactions can be written as [21, 28, 37]:

2 $$ 4{\text{Zn}}^{2 + } + {\text{SO}}_{4}^{2 - } + 5{\text{H}}_{2} {\text{O}} + 6{\text{OH}}^{ - } \to {\text{ZnSO}}_{4} \cdot 3{\text{Zn}}({\text{OH}})_{2} \cdot 5{\text{H}}_{2} {\text{O}} $$

3 $$ {\text{MnO}}_{2} + {\text{H}}^{ + } + {\text{e}}^{ - } \to {\text{MnOOH}} $$

4 $$ 2{\text{MnO}}_{2} + 2{\text{H}}^{ + } + 2{\text{e}}^{ - } \to {\text{Mn}}_{2} {\text{O}}_{3} + {\text{H}}_{2} {\text{O}} $$

Phases in regions **e**, **f**, **g**, and **h** in Fig. 4c, d marked by yellow dash can be identified as α-MnOOH, α-Mn_2_O_3_, α-Mn_2_O_3_, and α-Zn_x_MnO_2_, respectively, through fast Fourier transform (FFT) in Fig. 4e–h (detailed calculation procedures are given in Table S2–S4). From the TEM-EDS result, the Zn element can be found in both regions A and B and there is no S element in these regions (Fig. 4b), further confirming that zinc-ion inserts into the nanorods. Besides, the generation of BZSP and α-MnOOH indicates that H^+^ and Zn^2+^ participate in the reaction. From the above discussions, zinc ion insertion in α-MnO_2_ occurs around 1.4 V versus Zn^2+^/Zn to generate α-Zn_*x*_MnO_2_, and proton conversion reaction happens between 1.0 and 1.3 V in the cathode and leads to the generation of MnOOH, Mn_2_O_3_, and BZSP.

### Phase Evolution of Cathode in the First Charge Process

**Fig. 5:**
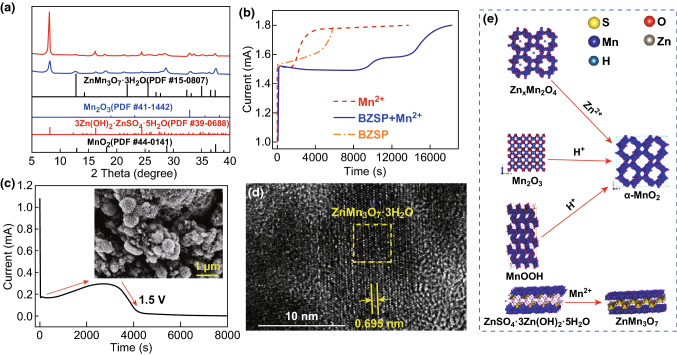
The first charge process: **a** XRD pattern of the fully charged cathode in the first charge process; **b** charging curves of α-MnO_2_ electrodes after discharge to 1 V in different cases; **c** constant voltage test; **d** HRTEM image and analysis result. **e** Phase evolution of cathode during the first charge process

To detect the phase evolution of MnO_2_ cathode during the charge process, the battery is discharged to 1.0 V and then charged to 1.9 V in 2 M ZnSO_4_ and 2 M ZnSO_4_ + 0.5 M MnSO_4_ electrolyte, respectively. The discharging products of Mn_2_O_3_, BZSP, and MnO_2_ still exist on the charged cathode, and besides, new phase ZnMn_3_O_7_·3H_2_O generates (Fig. 5a). It is worth noting that when adding Mn^2+^ in the electrolyte, the diffraction peaks of ZnMn_3_O_7_·3H_2_O become strong while the diffraction peaks of BZSP become weak (Fig. 5a). The morphology of cathode changes greatly (from hexagonal nanosheets to ball-like nanoflowers) during the initial charging process as shown in Figs. S7a–f and S8a–d. Figure S7a–f shows that BZSP exists in the 2 M ZnSO_4_ solution while almost disappears in 2 M ZnSO_4_ + 0.5 M MnSO_4_ solution during the charging process. It means that added Mn^2+^ in the electrolyte can promote the dissolution of BZSP. To confirm this, the fresh cathode was discharged to 1.0 V in 2 M ZnSO_4_ + 0.5 M MnSO_4_ solution and then soaked in potassium hydrogen phthalate solution (pH = 4.0) for 72 h to completely dissolve BZSP. The electrode without BZSP was then charged in 2 M ZnSO_4_ + 0.5 M MnSO_4_ electrolyte. Meanwhile, the discharged electrode with BZSP was separately charged in the electrolytes of 2 M ZnSO_4_ and 2 M ZnSO_4_ + 0.5 M MnSO_4_. The charge processes of the three conditions are shown in Fig. 5b. It can be seen that the platform, which means heterogeneous reaction happens, occurs at 1.5 V only when BZSP and Mn^2+^ both exist. To further confirm this, a discharged electrode with BZSP charge at 1.5 V in the 2 M ZnSO_4_ + 0.5 M MnSO_4_ solution. The nanoflower-like product generates (inset in Fig. 5c) as expected. ZnMn_3_O_7_·3H_2_O is also found in HRTEM in Fig. 5d. Combining with the result of XRD, the nanoflower is ZnMn_3_O_7_·3H_2_O. The dissolution of BZSP (nanosheets in Fig. S8a–d) and the occurrence of the new phase (nanoflowers in Fig. S8a–b) ZnMn_3_O_7_·3H_2_O indicate that ZnMn_3_O_7_·3H_2_O generate from the conversion reaction between BZSP and Mn^2+^. The nanoflower is Zn(OH)_2_ and MnO_2_ alternately layered structure as seen from Fig. S9. The conversion reactions are as follows: Zn^2+^ in the interlayer of Zn(OH)_2_ (Fig. S10) exchanges with Mn^2+^ and then the Mn^2+^ is chemically oxidized during the charging process. With continuous oxidation of Mn^2+^, the Mn^2+^ reacts with H_2_O to generate ZnMn_3_O_7_·3H_2_O (Fig. S10). The structure evolution of cathode in the first charge is shown in Fig. 5e. In other words, besides H^+^ and Zn^2+^, Mn^2+^ also participates in the reactions that occur during the discharge/charge process as the conversion reactions are supposed as follows:

5 $$ 3({\text{ZnSO}}_{4} \cdot 3{\text{Zn}}({\text{OH}})_{2} \cdot 5{\text{H}}_{2} {\text{O}}) + 3{\text{Mn}}^{2 + } + 8{\text{e}}^{ - } \to {\text{ZnMn}}_{3} {\text{O}}_{7} \cdot 3{\text{H}}_{2} {\text{O}} + 3{\text{ZnSO}}_{4} + 18{\text{OH}}^{ - } + 8{\text{Zn}}^{2 + } + 12{\text{H}}_{2} {\text{O}} $$

The generation of ZnMn_3_O_7_·3H_2_O during the first charge process can explain the phenomenon that the second discharge curve of the battery is different from the first discharge curve. In a word, during the first charging process, Zn_*x*_MnO_2_ and MnOOH reversibly become α-MnO_2_ with the extraction of Zn^2+^ and H^+^, while ZnMn_3_O_7_·3H_2_O acts as the host for the insertion of Zn^2+^ forms through the reaction between Mn^2+^ and BZSP.

### Phase Evolution of Cathode in Subsequent Discharge/Charge Processes

**Fig. 6:**
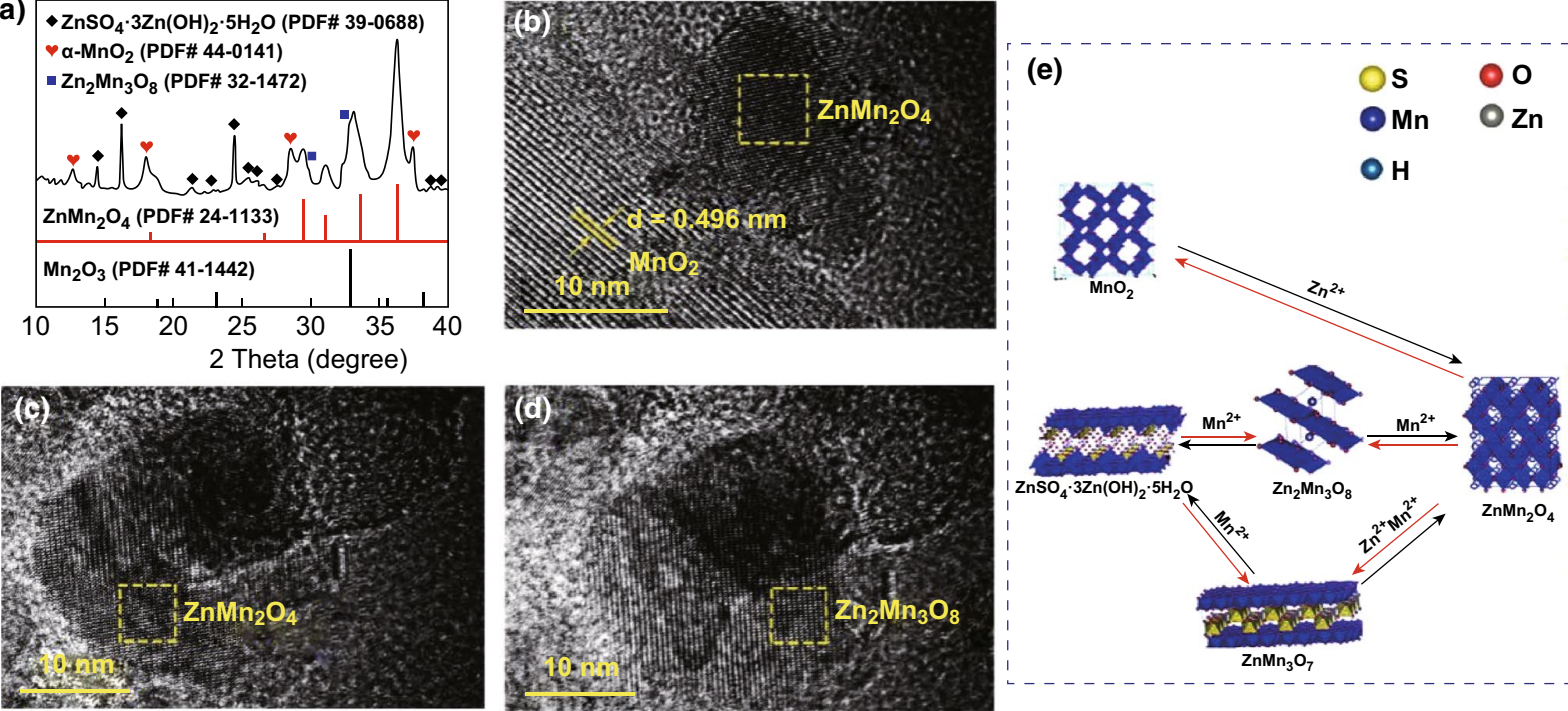
The MnO_2_ cathode after 100 charge/discharge cycles in 2 M ZnSO_4_ + 0.5 M MnSO_4_ electrolyte: **a** XRD patterns; **b**–**d** HRTEM images; **e** phase evolution of cathode during the repeated charge/discharge processes after the first charge/discharge cycle

The phases of the battery system after 100 charges/discharge cycles are also studied. XRD patterns of the discharged cathode after 100 cycles in 2 M ZnSO_4_ + 0.5 M MnSO_4_ solution in Fig. 6a imply that the main phases of the system are ZnMn_2_O_4_ (PDF#24-1133), Zn_2_Mn_3_O_8_ (PDF#32-1472), Mn_2_O_3_, BZSP, and α-MnO_2_. These components are confirmed in HRTEM images in Fig. 6b–d. As a contrast, the battery in the 2 M ZnSO_4_ solution, the main phases of the system are ZnMn_2_O_4_, ZnMn_3_O_7_, Mn_2_O_3_, and BZSP (Fig. S11a, b). ZnMn_2_O_4_ is on the surface of the α-MnO_2_ in HRTEM (Fig. 6b), so it may be converted from α-MnO_2_. Zn_2_Mn_3_O_8_ and ZnMn_2_O_4_ are coherent in the HRTEM (Fig. 6c, d). Thus, Zn_2_Mn_3_O_8_ reacts with Mn^2+^ to form ZnMn_2_O_4_. (This will be further analyzed in the Mn–Zn–O diagram in the following part.) There are two kinds of nanoparticles after 100 cycles (Fig. S12), which may be converted from BZSP and Mn^2+^ as nanosheets and nanoparticles surround each other. Combining the XRD result (Fig. 6a), the phase is Zn_2_Mn_3_O_8_. Thus, Zn_2_Mn_3_O_8_ is generated from the reactions between BZSP and Mn^2+^. The structure evolution of cathode after 100 cycles is shown in Fig. 6e. Reactions between them are as follows:

6 $$ {\text{Zn}}_{x} {\text{MnO}}_{2} + (0.5 - x){\text{Zn}}^{2 + } + (1 - 2x){\text{e}}^{ - } \to 0.5{\text{ZnMn}}_{2} {\text{O}}_{4} $$

7 $$ {\text{Zn}}_{2} {\text{Mn}}_{3} {\text{O}}_{8} + {\text{Mn}}^{2 + } \to 2{\text{ZnMn}}_{2} {\text{O}}_{4} $$

To sum up, within the continuous charge/discharge process, ZnMn_2_O_4_ and Zn_2_Mn_3_O_8_ as host for insertion of Zn^2+^ further generate on the surface of MnO_2_, which implies that the phase change of MnO_2_ cathode is irreversible.

### Thermodynamic Analysis

**Fig. 7:**
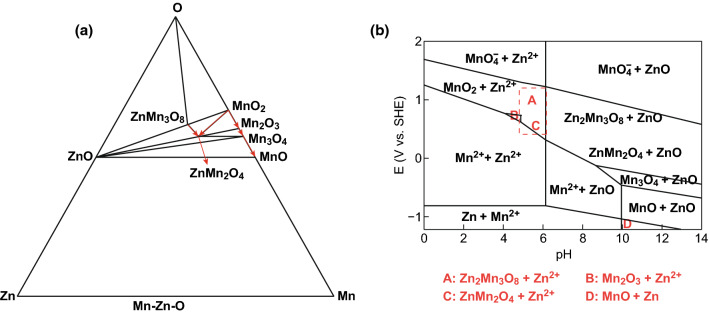
a Zn–Mn–O diagram and **b** E-pH diagram of Zn–Mn–H_2_O system

When dynamic conditions are met, the phases can be predicted from Zn-Mn–O diagram since the system of the Zn/ZnSO_4_ + MnSO_4_/MnO_2_ battery reaches an equilibrium state after a certain cycle. The isothermal cross section of the phase diagram (25 °C) is shown in Fig. 7a. Since MnO_2_ is used as an active material and ZnSO_4_ + MnSO_4_ as the electrolyte for the beginning, the phase of the reaction is bound to three elements (Mn, O, and Zn). When the thermodynamic stability of the system reaches, the definite phases include manganese oxide (MnO_2_, Mn_2_O_3_, Mn_3_O_4_, and MnO) and zinc manganese oxide (ZnMn_2_O_4_ and Zn_2_Mn_3_O_8_) (Fig. 7a). Detailed density functional theory calculation and theoretical analysis of MnO_2_ as a cathode of ZIBs are given in Discussion S1 and S2 in Supporting Information). There are two paths to the reduction of MnO_2_: (i) MnO_2_ → Mn_2_O_3_ → Mn_3_O_4_ → MnO, in which path there is no Zn involvement; (ii) MnO_2_ → ZnMn_2_O_4_ with Zn involvement. For Mn_2_O_3_ oxidation, it is directly oxidized to MnO_2_: Mn_2_O_3_ → MnO_2_. The oxidation of ZnMn_2_O_4_ has two paths: Zn extracts out completely from ZnMn_2_O_4_ to generate MnO_2_ or Mn partially removes from ZnMn_2_O_4_ to generate Zn_2_Mn_3_O_8_. They can express as: (1) ZnMn_2_O_4_ → MnO_2_; (2) ZnMn_2_O_4_ → Zn_2_Mn_3_O_8_. And there’s only one way for the Zn_2_Mn_3_O_8_ reduction: Zn_2_Mn_3_O_8_ → ZnMn_2_O_4_. In the Zn/ZnSO_4_ + MnSO_4_/α-MnO_2_ system studied in this paper, MnO dissolves in the electrolyte or can be inhibited when there is a certain concentration of Mn^2+^. Thus, there are five kinds of phases that may exist, such as MnO_2_, Mn_2_O_3_, Mn_3_O_4_, ZnMn_2_O_4_, and Zn_2_Mn_3_O_8_. When taking PH and potentials into consideration, MnO_2_, Mn_2_O_3_, ZnMn_2_O_4_, MnOOH, and Zn_2_Mn_3_O_8_ can form in our system but MnOOH is not stable, which matches well with our work (the red region in Fig. 7b). The reaction route is concluded to be as follows: (1) MnO_2_ → Mn_2_O_3_, (2) MnO_2_ → ZnMn_2_O_4_ and (3) Zn_2_Mn_3_O_8_ → ZnMn_2_O_4_ (Fig. 7a). MnO_2_, Mn_2_O_3_, ZnMn_2_O_4_, and Zn_2_Mn_3_O_8_ are stable phases and all can store Zn^2+^ (Fig. 7b).

Overall, we combined electrochemical analysis, phase identification with E-pH diagram of the Mn–Zn–H_2_O system together to analyze charge/discharge processes of aqueous rechargeable Zn//MnO_2_ batteries and revealed complicated phase evolution of the cathode (i.e., what new phases will form and how can they form in different charge/discharge stages). We obtained some different conclusions from previous literature. For example, Sun et al. thought that the conversion of H^+^ occurs before Zn^2+^ insertion [38]. But we find that Zn^2+^ insertion occurs before the conversion of H^+^ in the first discharge process, and this is confirmed by thermodynamic analysis. Besides, previous literature deemed that the disappearance of BZSP is always caused by the change in electrolyte pH [34], but we find that BZSP can react with Mn^2+^ in the electrolyte to form a new phase of ZnMn_3_O_7_.

## Conclusions

Based on experimental results and theoretical analysis of Zn/MnO_2_ ZIBs with the mixture electrolyte of ZnSO_4_ + MnSO_4_ aqueous solution, we found that the mechanism in ZIBs is dynamic and the phase transformation at MnO_2_ cathode is irreversible during charge/discharge processes. Not only H^+^ and Zn^2+^ but also Mn^2+^ in the electrolyte take part in the reactions. In the first discharge process, Zn_*x*_MnO_2_, MnOOH, Mn_2_O_3_, and by-product BZSP generate, and then in the first charge process, α-MnO_2_ and ZnMn_3_O_7_·3H_2_O appear. In the following charge/discharge processes, ZnMn_2_O_4_ and ZnMn_3_O_8_ are further generated on the surface of MnO_2_ and serve as the hosts for Zn^2+^ insertion. The mechanism becomes dynamic and complex because of the co-participation of the insertion process, conversion reaction, and oxidation reactions. The aforementioned phase changes inside ZIBs are well explained by the Mn–Zn–O phase diagram and the E-pH diagram. This work can provide guidance for continual research from the following aspects. (i) The research method combining electrochemical analysis and phase identification with E-pH diagram together can be used to analyze charge/discharge processes of other electrochemical energy storage systems, such as aqueous rechargeable Zn//V_2_O_5_ batteries. (ii) According to the proposed energy storage systems in this work, at least two approaches can be applied to enhance cycling performance of ZIBs: One is adding Mn^2+^ to promote the disappearance of BZSP, and the other one is adding pH buffer into the electrolytes or preparing solid electrolytes to prohibit the generation of OH– and BZSP.

## Electronic supplementary material

Below is the link to the electronic supplementary material.

Supplementary material 1 (PDF 2173 kb)
