# Supplementary material for: Novel Insights into Energy Storage Mechanism of Aqueous Rechargeable Zn/MnO2 Batteries with Participation of Mn2+
Source: Nanomicro Lett. 2019 Jun 6;11:49. doi: 10.1007/s40820-019-0278-9 (PMC7770901; doi:10.1007/s40820-019-0278-9)
Supplement: Supplementary file 1 — Supplementary material 1 (PDF 2173 kb) [file 40820_2019_278_MOESM1_ESM.pdf]

Supporting Information for

Novel Insights into Energy Storage Mechanism of Aqueous Rechargeable Zn/MnO_2 Batteries with Participation of Mn^{2+}

Yongfeng Huang[1, 2, #], Jian Mou[1, #], Wenbao Liu[1, 2], Xianli Wang[1], Liubing Dong[1, *], Feiyu Kang [1, 2, *], Chengjun Xu [1, *]

[1]Shenzhen Geim Graphene Center, Graduate School at Shenzhen, Tsinghua University, Shenzhen 518055, People's Republic of China

[2]State Key Laboratory of New Ceramics and Fine Processing, School of Materials Science and Engineering, Tsinghua University, Beijing 100084, People's Republic of China

[#] These authors contributed equally to this work

*Corresponding authors. E-mail: dong1060@126.com (L. Dong); fykang@mail.tsinghua.edu.cn (F. Kang); vivaxuchengjun@163.com (C. Xu)

Discussion S1 Density Functional Theory Calculation

Density functional theory (DFT) calculations were performed with the Vienna ab-initio simulation package with projector augmented wave pseudo-potentials. The Perdew-Burke-Ernzerhof exchange-correlation functional is used with the Hubbard U-corrections to correct the self-interaction error introduced by the highly localized *d* orbitals. In our calculations, the parameter of U-J=3.9 eV was employed. The cut-off for the plane wave basis set was 520 eV and a minimum of 3*3*5 gamma-centered k-point mesh was used. The calculated crystal structures were visualized using the program Visualization for Electronic and Structural Analysis. The relaxation of one Zn^{2+} into one α-MnO_2 cell was carried out. Before the DFT calculation, α-MnO_2 cell has been relaxed. Calculation results are in Table S5.

Discussion S2 Theoretical Analysis of MnO_2 as a Cathode in the Aqueous Zinc-ion Battery

The anode potential (ψ^-) is equal to the equilibrium potential of zinc dissolution and deposition. ψ^- = -0.763 V vs standard hydrogen electrode for the depth of discharge.

$a_{Zn^{2+}}$ = 0.14 (ion activity of Zn^{2+} is calculated, as shown in Eqs. S3-S5). The pH value of electrolyte is 5.5 (Fig. S13) [S1] at which $ZnSO_4 \cdot 3Zn(OH)_2 \cdot 5H_2O$ (noted as "BZSP") would be produced. There are six theoretical electrochemical reduction progresses of MnO_2 in Mn-Zn-H_2O system (Table S6). The cathode potential is calculated by the Nernst equation. The cathode potential descents continuously when the homogeneous reaction occurs. The cathode potential keeps constant when the heterogeneous reaction happens, while changes of ion activity and pH value are ignored. If the anode potential also keeps constant, there would be a platform in the constant voltage charge/discharge curve. These six reduction reactions differ from the theoretical reductive phases, the potential of the reaction or the shape of batteries' constant voltage charge/discharge curve. The reductive phase of active materials, the potential of the reaction and the shape of batteries' constant voltage charge/discharge curve are determined by experiment. As such, the discharge mechanism of MnO_2 as the cathode can be figured out.

$$\Delta E_f = E_{\alpha-Zn_xMnO_2} - xE_{Zn} - E_{\alpha-MnO_2} \qquad \text{(S1)}$$

$$E_f = \Delta G_f \,/\, zF \qquad \text{(S2)}$$

In which ΔG_f is Gibbs free energy change of the reaction, z is electron transfer number and F is Faraday constant. ΔG_f can be calculated by the data in Table S7. ΔE_f is the formation of energy calculated by DFT.

From the experimental evidence, the take-off potential of R_2 is about 1.5 V vs. Zn^{2+}/Zn and the battery voltage descents continuously, the take-off potential of R_1 is about 1.35 V and the battery voltage keeps nearly constant, α-Zn_xMnO_2 or $ZnMn_2O_4$ would be produced during R_2 and MnOOH or Mn_2O_3 would be generated during R_1.

Batteries' voltage would be lower than the theoretical electromotive force due to the polarization. Thus, combined with the shape of constant voltage charge/discharge curve and the reductive phase, it can be concluded that heterogeneous reaction would occur during R_1 of initial discharge progress and the discharge mechanism in R_1 is proton-electron conversion mechanism or manganese oxide conversion mechanism. It can be also concluded that homogeneous reaction occurs in R_2 and the discharge mechanism in R_2 is zinc ion insertion mechanism and the final reductive phase is $ZnMn_2O_4$. That is, the peak at 1.3-1.5 V vs Zn^{2+}/Zn in cyclic voltammetry (CV) curve indicates the zinc ion insertion reaction, while the peak at 1-1.3 V indicates the reaction in which proton participated.

The formation of BZSP, MnOOH, Mn_2O_3, Mn^{2+} (aq), α-Zn_xMnO_2 or $Zn_2Mn_4O_8 \cdot H_2O$ is detected by experiment. Among these, the formation of Mn^{2+} (aq) is due to the reduction or the disproportionation of trivalent manganese ion (manganese in

MnOOH or Mn_2O_3). The formation of Mn_2O_3 is not the result of manganese oxide conversion mechanism but the production of dehydration of MnOOH. Zinc ion insertion reaction results in α- Zn_xMnO_2 and α- Zn_xMnO_2 may transfer to $ZnMn_2O_4$. Thus, the following reactions may occur during the process as shown in 2.1:

S2.1 Reactions for Calculation of Pourbaix Diagrams

$$MnO_4^- + 4H^+ + 3e^- = MnO_2 + 2H_2O \quad (S3)$$

$$MnO_2 + H^+ + e^- = MnOOH \quad (S4)$$

$$2MnO_2 + Zn^{2+} + 2e^- = ZnMn_2O_4 \quad (S5)$$

$$2MnO_2 + ZnO + 2H^+ + 2e^- = ZnMn_2O_4 + H_2O \quad (S6)$$

$$MnO_2 + 4H^+ + 2e^- = Mn^{2+} + 2H_2O \quad (S7)$$

$$2MnOOH + Zn^{2+} = ZnMn_2O_4 + H_2O \quad (S8)$$

$$3MnOOH + 4H^+ + 4e^- + = Mn_3O_4 + 2H_2O \quad (S9)$$

$$MnOOH + 3H^+ + e^- = Mn^{2+} + 2H_2O \quad (S10)$$

$$MnOOH + H^+ + e^- = MnO + H_2O \quad (S11)$$

$$ZnMn_2O_4 + 4H^+ + 2e^- = Zn^{2+} + 2MnO + 2H_2O \quad (S12)$$

$$ZnMn_2O_4 + 8H^+ + 2e^- = Zn^{2+} + 2Mn^{2+} + 4H_2O \quad (S13)$$

$$Mn_3O_4 + 2H^+ + 2e^- = 3MnO + H_2O \quad (S14)$$

$$Mn_3O_4 + 8H^+ + 2e^- = 3Mn^{2+} + 4H_2O \quad (S15)$$

$$MnO + 2H^+ = Mn^{2+} + H_2O \quad (S16)$$

$$MnO + 2H^+ + 2e^- = Mn + H_2O \quad (S17)$$

$$Mn^{2+} + 2e^- = Mn \quad (S18)$$

S2.2 Calculation of Zinc-ion Activity

Ionic activity coefficient is calculated by Eq. S19:

$$\lg \gamma_i = \frac{-Az_i^{\,2}\sqrt{I}}{1 + BR_i^{\,H}\sqrt{I}} \qquad \text{(S19)}$$

in which γ_i is activity coefficient, I is ionic strength, z_i is in charage of the ion, R_i^H (angstrom) is hydrated ion size of the ion, A and B are coefficient (At 25 °C in aqueous solutions, A and B are 0.5115 and 0.3291, respectively).

I is calculated by Eq. S20:

$$I = \frac{1}{2}\sum c_b z_b^{\,2} \qquad \text{(S20)}$$

c_b is the molar concentration of ion b in solution. z_b is the charge of ion b in solution.

Zinc-ion activity is calculated by Eq. S21:

$$a_{Zn^{2+}} = \gamma_{Zn^{2+}} \cdot c_{Zn^{2+}} \qquad \text{(S21)}$$

In 2 mol L^{-1} $ZnSO_4$ solution, $I = 8$, $R_{Zn^{2+}}^H = 4.3$ angstrom^2, $R_{Mn^{2+}}^H = 4.38$ angstrom

$c_{Zn^{2+}}=2$, $z_{Zn^{2+}} = 2$. So, $\gamma_{Zn^{2+}} = 0.07$ $a_{Zn^{2+}} = 0.14$

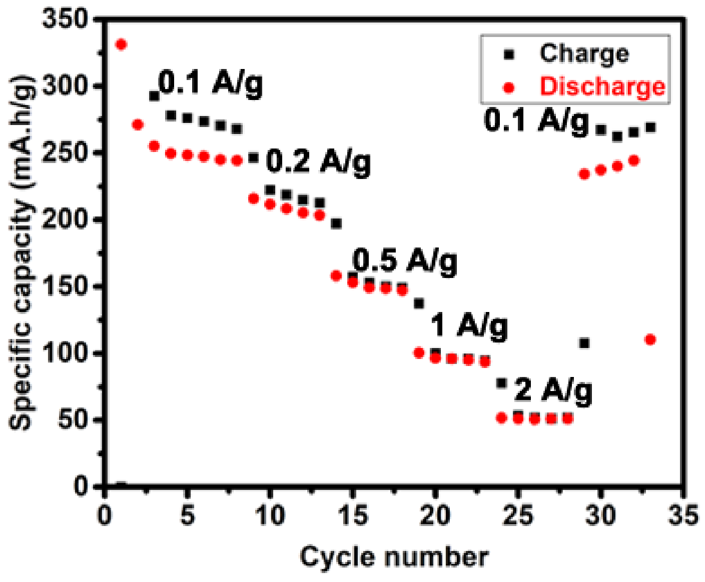

Fig. S1 Rate capabilities and cycling performances of the battery in 2 M $ZnSO_4$ + 0.5 M $MnSO_4$ system

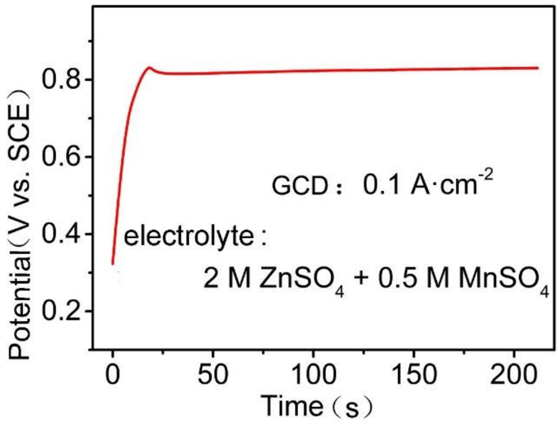

Fig. S2 Potential *vs.* saturated calomel electrode (SCE) of the electro-deposition of Mn^{2+} in 2 M $ZnSO_4$+0.5 M $MnSO_4$ at a constant current of 0.1 A cm^{-2}

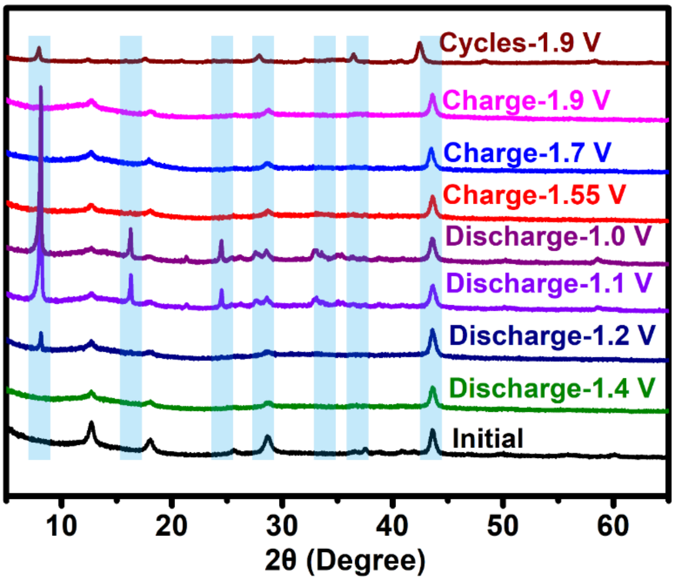

Fig. S3 XRD of the cathodes at different charge/discharge states

When the cathode is discharged to 1.4 V, no new phase produces. Nevertheless, characteristic peaks of the MnO_2 active material such as the peaks at 28° and 44° shift, which is attributed to the change of the layer spacing of MnO_2. When the battery is further discharged to 1.2 and 1.0 V at constant current, several new diffraction peaks appear and then become stronger, especially the peaks at 8° and 16 °, implying the generation of new phases. In charging process, some diffraction peaks cannot be detected, which means the disappearance of some phases. After 100 charge/discharge cycles, the XRD pattern is not in conformity with the XRD patterns of the cathode at original state and fully charged state in the 1st charge process (*e.g.*, the diffraction peaks at around 36° and 44°).

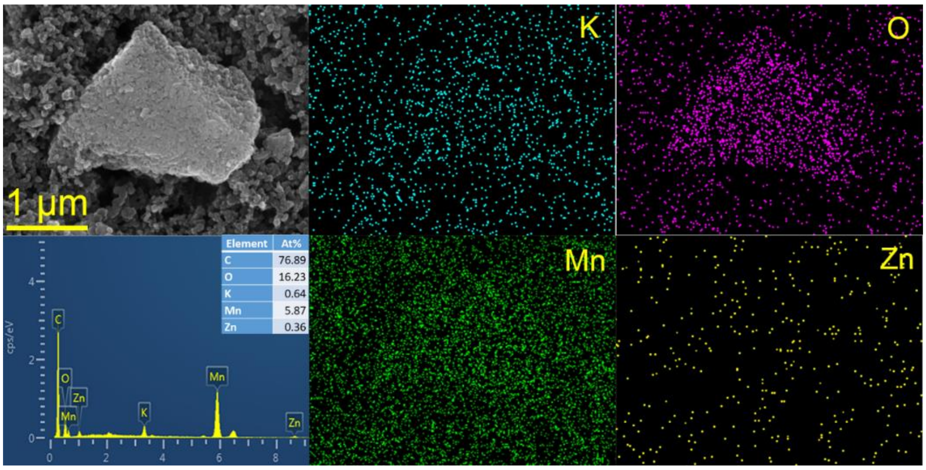

Fig. S4 SEM- EDS and mapping result of the cathode which was constant current discharge to 1.4 V and constant voltage discharge at 1.4 V for 2 h

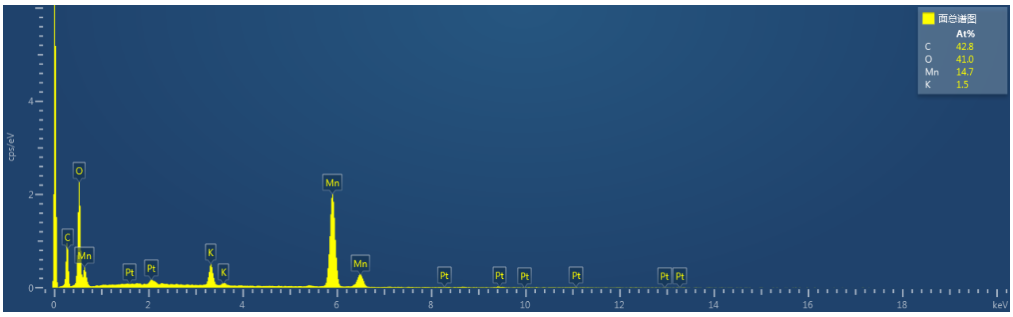

Fig. S5 SEM-EDS result of the cathode which has been immersed in the electrolyte for 2 days before cleaned with deionized water

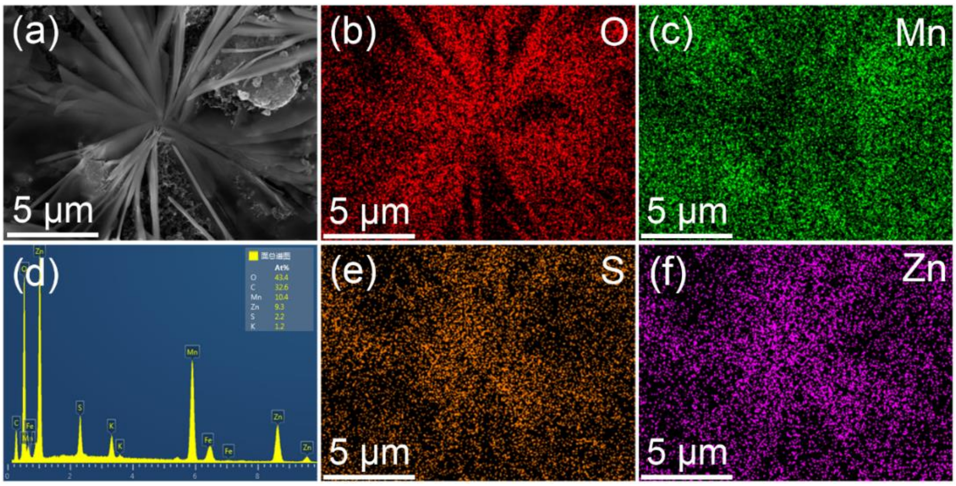

Fig. S6 SEM-EDS mapping of the region in which the hexagonal plates have a radial distribution. **a** SEM image. **b, c, e, f** The distribution of O, Mn, S, and Zn. **d** EDS spectrum

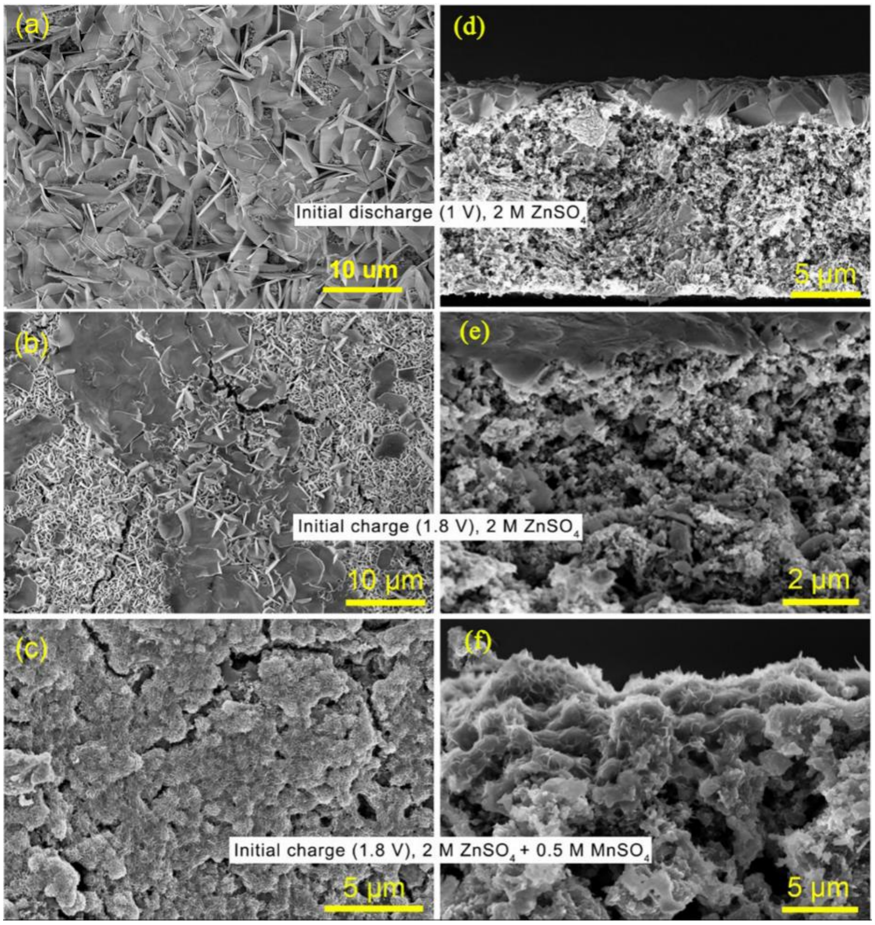

Fig. S7 SEM results of α-MnO_2 electrodes in different states, **a-c** the images of plane SEM images and **d-f** the section SEM image: **a**, **d** initial discharge; **b, e** initial charge in solution not added Mn^{2+}; **c, f** initial charge in solution added Mn^{2+}

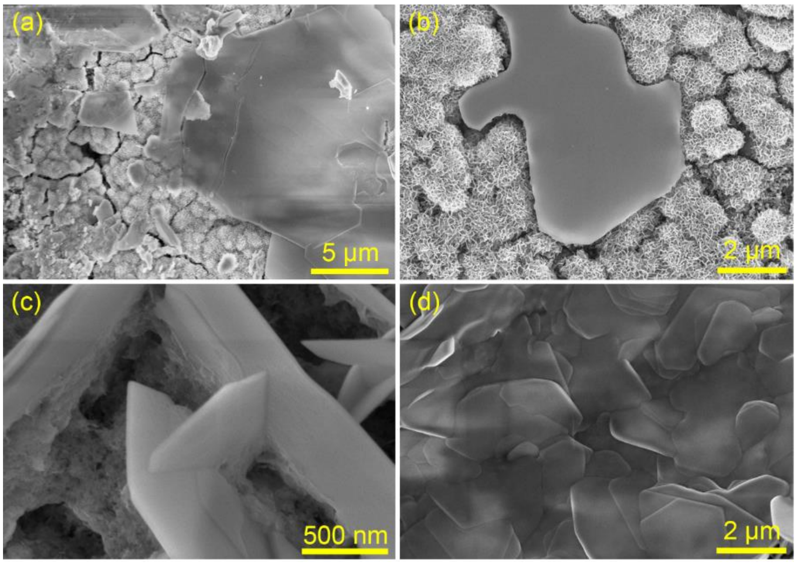

Fig. S8 SEM of dissolution of BZSP in the initial charge process

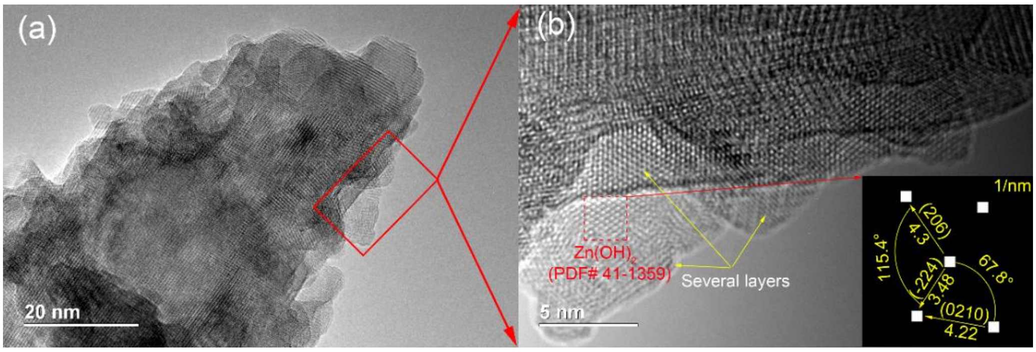

Fig. S9 a TEM **b** high-resolution TEM (HRTEM) images. Element content in electrodes and electrolytes was analyzed by inductively coupled results of nanorods after 1.5 V constant voltage charge

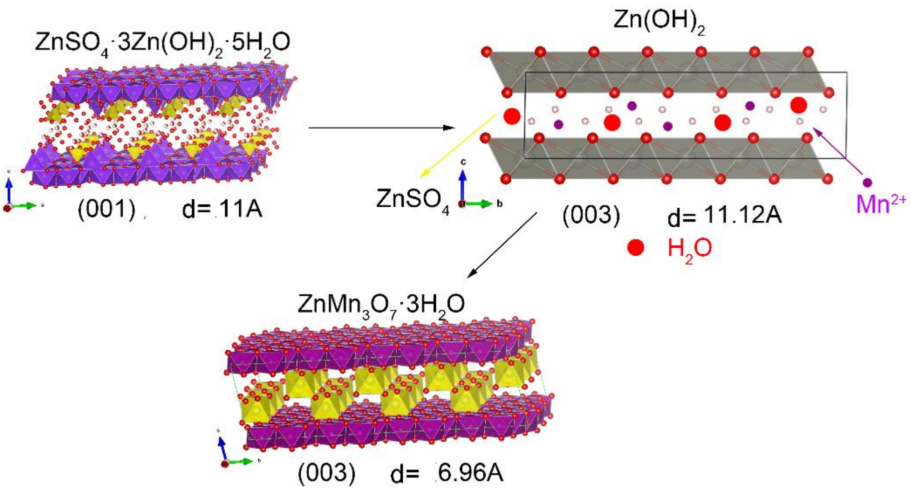

Fig. S10 Schematic diagram of transitions between BZSP and $ZnMn_3O_7{\cdot}3H_2O$

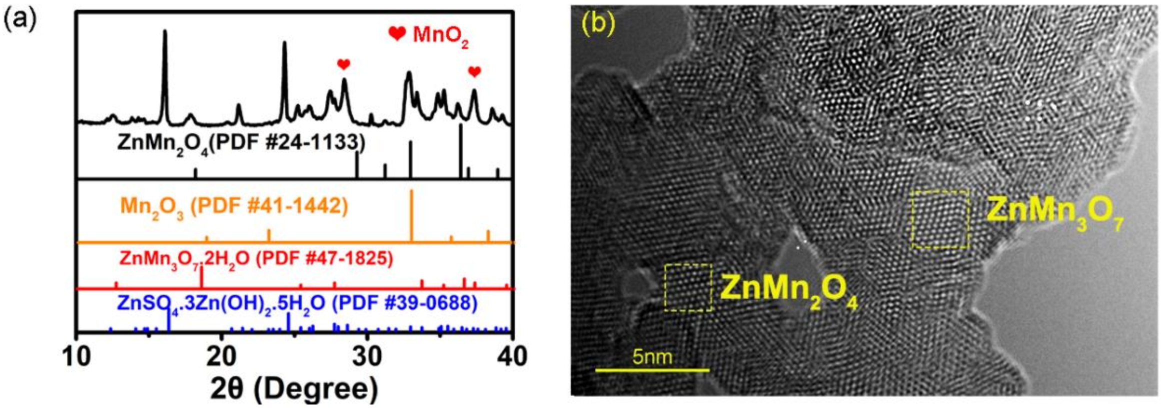

Fig. S11 The cathode after 100 charges/discharge cycles in 2 M $ZnSO_4$ electrolyte: **a** XRD pattern and **b** TEM image

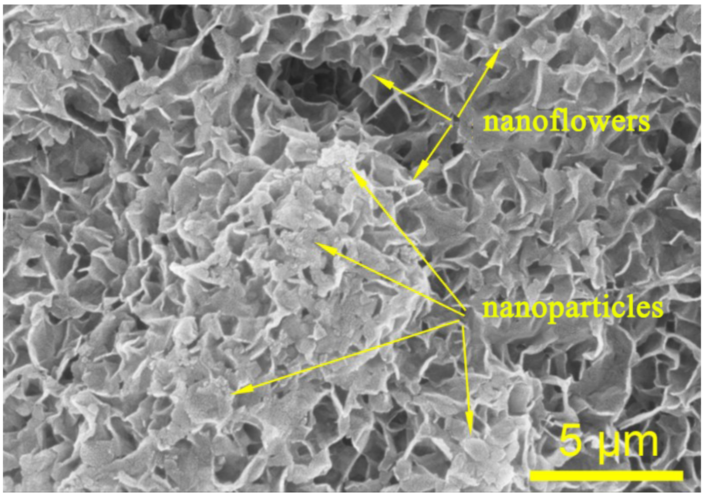

Fig. S12 SEM images of the structure generated during the process of the cycle

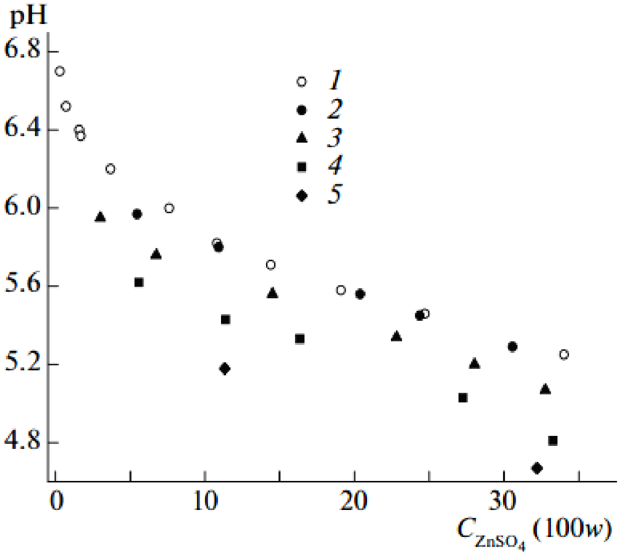

Fig. S13 The relationship between the concentration of $ZnSO_4$ and pH of the solution in the $ZnSO_4$ -$Zn(OH)_2$ - H_2O system at (1, 2) 291.15, (3) 298.15, (4) 308.15, and (5) 323.15 K. The figure is copied from Wu's work [S2]

Table S1 Plasma atomic emission spectrometry (ICP-AES) results

Sample	Element content		
	Mn (ppm)	K (ppm)	Zn (ppm)
1. the original cathode	37.2	4.6	--
2. the cathode constant-current discharged from OCV to 1.0 V	23.4	2.3	63.2
3. the cathode constant-voltage discharged at 1.4 V for 4 hours	36.7	2.4	2.9
4. the electrolyte with sample 2	12.4	2.1	12950

Table S2 Calculation procedures of fast Fourier transform (FFT) in Fig. 4e

	**α-MnOOH (PDF #24-0713)**			**Experiment**		
	Face	d (Å)	Angel (degree)	Length (nm)	Face	Angel (degree)
FFT of 4e	{120}	3.4715	<(021),(101)>=41.77	a=1/0.3612	(1-20)	∠A=39.62
	{101}	2.4288	<(021),(1-20)>=107.86	c=1/0.2369	(101)	∠C=76.36
	{021}	2.5294	<(101),(1-20)>=66.08	b=1/0.2487	(021)	∠B=64.02

Table S3 Calculation procedures of FFT in Fig. 4f-g

	**Mn_2O_3 (PDF #41-1442)**			**Experiment**		
	Face	d (Å)	Angel (degree)	Length (nm)	Face	Angel (degree)
FFT of 4f	{222}	2.7160	<(2-2-2),(400)>=54.73	a=1/0.2729	(222)	∠A=55.46
	{400}	2.3540	<(2-2-2),(222)>=109.47	c=1/0.2412	(400)	∠C=77.01
	{222}	2.7160	<(400),(222)>=54.74	b=1/0.2713	(2-2-2)	∠B=55.42
FFT of 4g	{222}	2.7160	<(321),(14-1)>=50.95	a=1/0.2719	(2-22)	∠A=52.07
	{321}	2.5140	<(2-22),(14-1)>=122.98	c=1/0.2546	(321)	∠C=57.23
	{411}	2.2194	<(2-22),(321)> =72.02	b=1/0.2306	(14-1)	∠B=70.70

Table S4 Calculation procedures of FFT in Fig. 4h

	**α-MnO_2 (PDF #44-0141)**			**Experiment**		
	Face	d (Å)	Angel (degree)	Length (nm)	Face	Angel (degree)
FFT of 4h	{310}	3.0930	<(211),(3-21)>=39.39	a=1/0.3083	(-130)	∠A=39.62
	{321}	1.9699	<(-130),(3-21)>=124.95	c=1/0.2440	(211)	∠C=57.02
	{211}	2.3950	<(-130),(211)>=85.56	b=1/0.2024	(3-21)	∠B=83.36

Table S5 Calculation results of inserting one Zn^{2+} into one α-MnO_2 cell

Site	Wyckoff Position	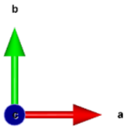	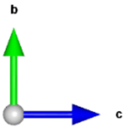	a (Å)	b (Å)	c (Å)	ΔV (%)	ΔE (eV)
2a	(0.5,0.5,0.5)	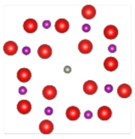	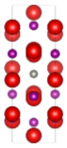	9.71	9.71	3.01	-1.13	-1.23
2b	(0.5,0.5,0)	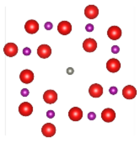	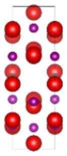	9.93	9.93	2.93	0.78	-0.2
8h	(0.6,0.5,0)	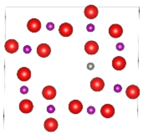	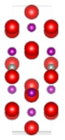	10.41	9.56	2.95	2.44	-1.58
8h'	(0.6,0.5,0.5)	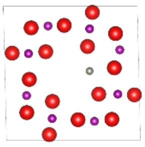	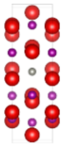	10.02	10.08	2.96	4.01	-1.34

Table S6 Six possible electrochemical reduction routes of MnO_2 as cathode Zn-Mn-H_2O system

**Mechanism**	**Reaction in cathode**	**Reaction type**	**Cathode Potential (V *vs. SHE*)**	**E (V)**
Proton-electron conversion	$MnO_2 + H^+ + e^- = MnOOH$	Heterogeneous reaction	$\psi^+ = 1.036 - 0.0592pH$	1.47
Proton-electron	$MnO_2 + xH^+ + xe^- = H_xMnO_2$	Homogeneous reaction	$\psi^+ = E - \psi$	$E = -\Delta E_f / e$
Manganese oxide conversion	$2MnO_2 + 2H^+ + 2e^- = Mn_2O_3 + H_2O$	Heterogeneous reaction	$\psi^+ = 0.974 - 0.0592pH$	1.41
Mn^{2+}	$MnO_2 + 4H^+ + 2e^- = Mn^{2+} + 2H_2O$	Heterogeneous reaction	$\psi^+ = 1.23 + 0.03\log a_{Mn^{2+}} - 0.12pH$	Determined by $a_{Mn^{2+}}$ and pH
Zinc ion insertion conversion	$Zn^{2+} + 2MnO_2 + 2e^- = ZnMn_2O_4$	Heterogeneous reaction	$\psi^+ = 0.76 - 0.03\lg a_{Zn^{2+}}$	1.52

Zinc ion insertion	$xZn^{2+} + MnO_2 + 2xe^- = Zn_xMnO_2$	Homogeneous reaction	$\psi^+ = E - \psi^-$	$E = \Delta E_f / e1.58$ $(x = 0.125)$

Table S7 ΔG data of some possible species in Zn-Mn-H_2O system

Species	ΔG (kcal mol^{-1})*
H_2O	-56.678
Mn^{2+}	-54.493
MnO	-86.719
$Mn(OH)_2$	-147.176
Mn_3O_4	-306.654
MnOOH	-135.547
Mn_2O_3	-210.557
$ZnMn_2O_4$	-293.439
MnO_2	-111.65
Zn^{2+}	-35.194
ZnO	-76.571
$Zn(OH)_2$	-132.309
MnO_4^-	-106.836
Zn	0
Mn	0

* $T = 298.15$ K; $p = 1$ bar; data from the HSC Chemistry software.

Supplementary References

[S1] H. Pan, Y. Shao, P. Yan, Y. Cheng, K.S. Han et al., Reversible aqueous zinc/manganese oxide energy storage from conversion reactions. Nat. Energy **1**, 16039 (2016). https://doi.org/10.1038/nenergy.2016.39

[S2] X. Wu, X. Yin, Z. Chen, X. Yu, D. Zeng, Y. Tan, Experimental determination and model simulation of the solid-liquid equilibria in the $ZnSO_4$-$Zn(OH)_2$-H_2O system. Russ. J. Phys. Chem. A **89**, 958-962 (2015). https://doi.org/10.1134/S0036024415060345
